# Incidence of Physical Injuries in a Rural Community in Sri Lanka: Results of the First Community Survey in Sri Lanka

**DOI:** 10.4103/0970-0218.43229

**Published:** 2008-10

**Authors:** MD Lamawansa, A Piyathilake

**Affiliations:** Department of Surgery, University of Peradeniya, Sri Lanka

**Keywords:** Community incidence, injury incidence, intentional injuries, physical injuries, Sri Lanka, unintentional injuries

## Abstract

**Background:**

Injuries account for approximately 11% of all hospital admissions in Sri Lanka. However, no published data are available with regard to the community incidence of injuries in Sri Lanka.

**Objectives:**

To determine the community incidence of major intentional and unintentional physical injuries in a rural community in Sri Lanka.

**Materials and Methods:**

A rural community consisting of 225 families with 1029 inhabitants was studied. Data on major injuries for a period of one year were collected retrospectively.

**Results:**

There were 85 major injuries in the community during the year of study. This gives a major injury incidence of 82.6 per 1000 person years. This is three times the incidence based on hospital-derived data. Animal bites being the most common cause of injury was noted in 2.3% of the population followed by falls in 1.6%, contact with objects in 1.5%, cut injuries in 1% and road trauma in 1%.

**Conclusions:**

This study shows a higher incidence of major physical injuries (both intentional and unintentional) in the community than figures derived from hospital data. The prevention of injuries in a community such as the one studied here should be aimed at animal bites, falls, contacts with objects, cut injuries and road trauma.

## Introduction

Injuries are a major health problem encountered in low socioeconomic countries, including Sri Lanka.(1,2) Approximately 11% of all admissions to state sector hospitals in Sri Lanka are associated with some form of physical injuries (both intentional and unintentional).(2) Further, injuries remain the main cause of deaths among the people of the most active age group, i.e., from 25 to 49 years of age.(2)

In Sri Lanka, the currently available injury data are hospital derived. However, it is known that the hospital data show only a fraction of the complete picture of physical injuries in a community.(3–6) This is likely to be true for Sri Lanka since most of the injured are treated as outpatients. There are no published reports with regard to the community incidence of physical injuries in Sri Lanka. The present study aimed to estimate the community incidence of both intentional and unintentional physical injuries in a rural community in Sri Lanka.

## Materials and Methods

This was a descriptional retrospective community-based study. We studied two small adjoining villages in the Kadugannawa area in the Kandy district. These villages are Maligatenna and Mamudawela Gramasewa Niladari areas in the Yatinuwara Divisional Secretariat division of the Kandy district. The relevant authorities and community leaders of the area were informed about the study. This enabled us to conduct an introductory presentation to the villagers about the study and its purpose and methodology. The study was performed between August and October 2005. We included all 225 families residing in the two villages in the study. For the purpose of easy reference, the term ‘injury’ in the present article comprises both intentional and unintentional injuries.

The second author visited the families with the help of the Grama Sevaka (government representative) of the area and the main occupant or the spouse in each household was interviewed. A verbal informed consent was obtained prior to the commencement of the data collection. In the consent, a detailed explanation was provided about the study method and privacy of the data; moreover, it was explained to the participants that there will not be any discrimination under any condition if they do not want to participate in the study. Data were collected using a structured questionnaire. The type of data included the sociodemographic factors, type, cause and place of major physical injuries (both intentional and unintentional), affected part of the body, type of treatment received, loss of work, disability related to injury. Data were collected on all family members. Major injuries were defined as those that resulted in work loss of greater than 1 day, hospitalization for greater than 24 h irrespective of the service provider or caused significant disability. Disability was defined as a temporary loss of function for more than one week or permanent loss of function (partial or complete) of part of the body. In deciding on the level of education, children below 12 years were pooled with the level of education of their mothers.

The participants were requested to recall major injuries for a period of one year preceding the interview and injury-related deaths for five years. Since there were repetitive injuries in three people, injury events were considered for calculations and not the number of people injured. The effect of injuries on the family is described as the loss of number of person days due to hospitalization or confinement to bed.

## Results

The village under study comprised 225 families with a total population of 1029. Of these, 166 were children below 12 years, 429 were adult males and 434 were adult females. Of the population studied, 14.9% (154) belonged to social classes 1 and 2, 39.9% in social classes 3 and 4 and 42.3% (435) in class 5. No formal education was reported in 164 (15.9%) individuals (we considered the education of the children below 12 years to be the same as that of their mothers). There were 512 (49.7%) individuals who had completed school education up to the ordinary level examination and 351 (34%) beyond the O level.

There were 85 major injuries for the preceding one year and no injury-related death for the preceding five years. These 85 injuries occurred in 82 individuals; of these, 18 were children, 41 were males and 23 were females. Table 1 presents the age distribution in the general population and the number of injuries in each age group. Tables 2, 3 and 4 present the cause of injuries, the site of injuries and the institution/place where treatments were administered.

**Table 1:** Age distribution of the study population and number of injuries in each age group

Age groups (years)	Number of people in the community n = 1029 (%)	Number of people injured in each age group (%)	SE*	95% CI†	Number of injuries in each age group a percentage of total number of injuries (n = 85)
<12	166 (16)	13 (7.8)	2.08	3.73, 11.97	15.3
12.1–25	222 (22)	10 (4.5)	1.39	1.78, 7.22	11.8
25.1–45	350 (34)	34 (9.7)	1.58	6.60, 12.80	(40)
45.1–60	169 (16)	21 (12.4)	2.53	7.44, 17.36	(24.7)
<60.1	122 (12)	7 (5.7)	2.09	1.59, 9.81	(8.2)
Total	1029 (100)	85 (8.2)	0.85	6.52, 9.88	(100)

* Standard error of age specific injury proportions

† 95% confidence interval (CI) calculated for each age specific injury proportions

**Table 2:** Place where injury occured

	Places where injury occurred (% of total injuries) n = 85	Places where injury occurred (% of the total population) n = 1029	SE*	95% CI†
Home	29 (34)	2.8	0.51	1.83, 3.85
Road	35 (41.1)	3.4	0.56	2.30, 4.50
Factory	12 (14)	1.2	0.34	0.53, 1.87
Recreational	4 (6)	0.4	0.20	0, 0.78
School	2 (2.4)	0.2	0.14	−0.08, 0.46
Farm	3 (3.5)	0.3	0.17	−0.04, 0.62

* Standard error of the places where injuries occurred as a proportion of the total population

† 95% confidence interval (CI) calculated for places where injuries occurred for the total population

**Table 3:** Causes of major injuries

	Causes of injuries (% of total injuries) n = 85	Causes of injuries (% of the total population) n = 1029	SE*	95% CI†
Animal bites	24 (28.2)	2.3	0.47	1.40, 3.21
Falls	16 (18.8)	1.6	0.39	0.79, 2.33
Objects fallen on the victim	15 (17.6)	1.5	0.38	0.72, 2.20
Cut injuries	10 (11.8)	1.0	0.31	0.30, 1.67
Road trauma	10 (11.8)	1.0	0.31	0.30, 1.67
Thorn pricks	5 (5.9)	0.5	0.21	0.06, 0.92
Burns	3 (3.5)	0.3	0.17	−0.03, 0.62
Assaults	2 (2.4)	0.2	0.14	0.08, 0.46

* Standard error of the causes of injuries as a proportion of the total population

† 95% confidence interval (CI) calculated for causes of injuries for the total population

**Table 4:** The types of medical care received

Type of treatment	Total number of injuries n = 85
Local hospital	24 (28.2%)
Tertiary care	18 (21.2%)
Indigenous	10 (11.8%)
Home remedies	13 (15.3%)
Private institutions	20 (23.5%)

**Figure 1:**
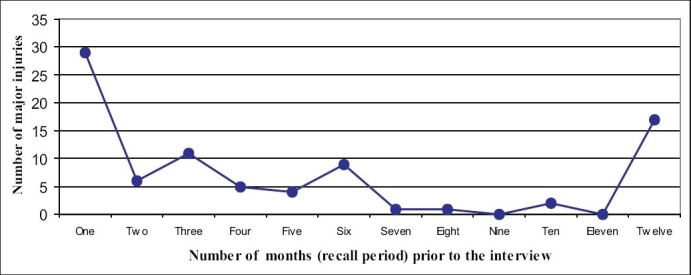
Time (monthly) distribution of major injuries

Of the 85 major injuries, 29 were reported to have occurred during the one month period preceding the interview and only 39 occurred during the second to 11 preceding months and 17 in the preceding 12^th^ month [Figure 1]. The total duration of the inpatient treatment for all injuries was 187 days. The total days of work loss due to injuries was 1487 days. Some form of partial permanent disability was observed in 19% (16 of the 85 individuals) of the injured individuals. None had total permanent disability.

## Discussion

Community surveys on injury incidence have several advantages. All injuries could be ascertained accurately only by performing community surveys. Since data are collected on all injuries regardless of the severity, place or type of treatment received, the characterization of injuries based on parameters such as demographic subpopulations and sociocultural subgroups are possible. Therefore, high-risk individuals could be identified and preventive measures instituted and evaluated as pattern of injuries and their causes could vary from one community to another.(6,7) Knowing the true incidence enables identification of the magnitude of the economic impact to the society incurred by the treatment costs and loss of work. Utilization of health care facilities too could be best evaluated by using community-based data. Further, community-based data permit direct comparison of injury rates among different communities. Although physical injuries are the leading cause of hospitalization in Sri Lanka, there are no published reports of community incidence of injuries in Sri Lanka. The present study was undertaken to address this problem.

Two small villages in close proximity to Colombo-Kandy highway was selected for the convenience of access in the evenings to visit for interviewing the chief occupant of the house. Since the two villages are located close to a main highway, many facilities are within the easy reach. The methodology and definitions used in the present study were adapted from World Health Organization (WHO) guidelines on conducting community studies on incidence of injury and similar studies that have been conducted in other countries.(5,7–9) The sample size was relatively small, consisting of only 225 households with 1029 occupants. Consequently, for further analysis of the causes and places of injuries [Tables 2 and 3], the numbers in some subgroups were too small to draw a significant conclusion. However, this is the first study of this kind, and therefore, could be used as a reference for conducting further studies in different communities in Sri Lanka.

## Incidence of injuries

During the study year, 85 major injuries were noted among the total study population (1029). This equals to 82.6 injuries per 1000 person years, which is approximately three times higher than the injury incidence calculated for Sri Lanka on hospital-derived data; in the year 2002 in Sri Lanka, the incidence was 27.37 injuries per 1000 person years.(2) Similar high prevalence has been reported in other countries with low socioeconomic condition; the reported incidence rates could be as high as 300 injuries per 1000 person years.(9,10) However, a parallel should be established carefully between the results of the present study and the results from other studies since the incidence among community studies are subject to methodological bias. The results may be affected due to the inclusion of minor injuries, socioeconomic factors, influence of seasonal variation and underestimation due to recall bias.

It is useful to examine the possible effect of recall bias on our results. In our study, of the 85 major injuries, 29 were reported during the month preceding the interview and only 56 in the remaining year. This appears to be possible cause/s for this difference and needs to be focused upon. In community studies of this nature, it has been shown that less severe injuries are often neglected especially if they are non-road-traffic injuries and if the disability period is less than seven days.(9,11) In one study involving a large population of 21105 in Ghana, Mock *et al.* reported that the estimated injury incidence lowered by 72% from a 1-month recall period to 12-month recall period.(9) The recall incidence is not affected by the age, gender, education or the type of the respondent. However, the recall bias may not be the only factor to have affected our results; there should have been a gradual decrease in the incidence with the increasing recall period. However, the incidence of injury shows three peaks beyond the first month [Figure 1]. The three peaks of high incidence noted at the 3, 6 and 12 recall months may indicate a convenient round time points that the responders could recall the incidents for.

## Incidence of injuries in different age groups

The highest number of injuries (40%) in our study was noted in the age group of 25–40 years although when calculated as the percentage of the population of this age group, it constitutes only 9.7% and is less than the incidence in the next higher age group (12.4%) [Table 1]. The elderly and young adults had fewer injuries and the incidences were 5.7% and 4.5%, respectively, in their age groups. These finding is in keeping with the national inpatient data that has shown the highest number of injuries to be observed among the people of the most active age group.(2) In general, the victims of these age groups are the sole bread winners of their respective families, and therefore, pose an additional economic burden.

## Place and cause of injuries

The most common cause of injuries was animal bites. This accounts for 29% of all injuries. Barring one, all injuries were due to dog bite. Although 41% of the reported injuries occurred on the road [Table 2], only 11.8% could be categorized as related to traffic accidents [Table 3]. The next two common causes of injuries were falls and objects fallen on the victim. Being a rural area, it is expected to find more animal bites and falls and less traffic-related accidents.

## Type of treatment received

Nearly half of the injured individuals attended a government run medical care facility. Local hospital and tertiary care facility in [Table 4] and 24% patients have taken in-hospital treatment. If we follow the hospital-derived data to study the incidence of injuries, only the latter 24% would be included since the national statistics compiled by the Ministry of Health does not include the patients treated at other institutions. Of all major injuries, therefore, 76% would go unnoticed.

The total duration of inpatient treatment for all injuries was 187 days (for a population of 1029 over a period of one year). This equals to 181.7 days of inpatient treatment per year per 1000 population. The total days of work loss due to injuries was 1487 days. Therefore, the annual loss of human work days would be 1445 per year per 1000 persons. Both these facts highlight the increased burden and the economic impact of injuries in a rural community in Sri Lanka. However, there is no data available for the country for significant comparison with different communities.

## Conclusions

This study, being the first community survey for injuries in Sri Lanka, results in the incidence of major injuries in a rural community in Sri Lanka. The incidence is at least three times the incidence based on hospital-derived data. The work loss and economic burden of injuries also appear to be high and need further evaluation.

High-risk groups and causes that were identified could be the targets for injury prevention. Prevention in the community such as the one studied should be aimed at dog bites, falls, injuries related to contact with objects, cuts and road trauma. Although community prevention methods could be instituted based on the present data, further studies are required to assess the injury incidence in different communities in Sri Lanka. Since there might be gross underreporting of major injuries, it is essential that the recall bias be quantified in the community. In future studies, major injuries may be recalled for a time period shorter than one year in order to improve the accuracy of injury incidence.
